# Monitoring ZEO apoptotic potential in 2D and 3D cell cultures and associated spectroscopic evidence on mode of interaction with DNA

**DOI:** 10.1038/s41598-017-02633-z

**Published:** 2017-05-31

**Authors:** Fahimeh Salehi, Hossein Behboudi, Gholamreza Kavoosi, Sussan K. Ardestani

**Affiliations:** 1 Institute of Biochemistry and Biophysics, Department of Biochemistry, University of Tehran, Tehran, Iran; 2 Institute of Biotechnology, Shiraz University, Shiraz, Iran

## Abstract

Recognizing new anticancer compounds to improve Breast cancer treatment seems crucial. Essential oil of *Zataria Multiflora* (ZEO) is a secondary metabolite with some biological properties, yet underlying cellular and molecular anticancer properties of ZEO is unclear. GC/MS analysis revealed that carvacrol is the major ingredient of the essential oil. ZEO increasingly suppressed viability in MDA-MB-231, MCF-7 and T47D Breast cancer cells while nontoxic to L929 normal cells in monolayer cell cultures (2D), whereas MDA-MB-231 multicellular spheroids (3D) were more resistant to inhibition. ZEO significantly induced cell apoptosis confirmed by fluorescent staining, flow cytometry analysis and DNA fragmentation in MDA-MB-231 2D and 3D cell cultures. ZEO increased ROS generation and subsequent loss of ΔΨm, caspase 3 activation and DNA damage which consequently caused G1 and G2/M cell cycle arrest in a dose- and time-dependent manner in 2D. S phase arrest occurred in cell spheroids therefore ZEO possible DNA interaction with gDNA was investigated and revealed ZEO binds DNA via intercalation. Altogether, these data corroborate anticancer properties of ZEO and suggest that cell culture format (2D monolayer vs. 3D spheroid) plays a critical role in drug response and provides new insights into the mechanisms underlying ZEO cytotoxicity effect on Breast cancer cells.

## Introduction

Breast cancer is a common type of malignancy among women and has a complex and heterogeneous nature. Due to its various underlying cellular and molecular characteristics this disease is indeed a collection of diseases with variable clinical behaviors and outcomes that makes its treatment quite challengeable with existing therapeutic procedures^1–3^. Currently, chemotherapy is the dominant and most effective cancer treatment. The main goal of chemotherapy is to impose death upon cancer cells via inducing apoptosis without triggering inflammatory response and with minimal side effect on normal cells. Although chemotherapy has succeeded to some degrees and responded well in certain types of tumors but in many cases it is unable to remove all cancer cells and may cause collateral damage to normal cells and tissues. Some synthetic compounds can prevent, suppress or even reverse the progression of cancer at the cost of adversely affecting rapidly dividing normal cells, increasing drug resistance and high treatment costs. Hence such drawbacks are necessary to be addressed to raise the success rate of chemotherapy treatment^4–6^. *In vitro* and *in vivo* studies on phytochemicals, a large group of plant products classified as alkaloids, saponins, glycosides, triterpenes and polyphenols have shown very promising anticancer properties^7^. Vinblastine, Vincristine and Taxol (tubulin-binding agent), teniposide and etoposide (topoisomerase II inhibitor) irinotecan and topotecan (topoisomerase I inhibitor), are traditional examples of plant-derived compounds with diverse applications in cancer therapeutics^6–9^. *Zataria Multiflora Boiss* also called Avishan-e-Shirazi in Iran is a thyme-like and aromatic traditional medicinal plant that belongs to the Lamiacea family and grows extensively in southern and central parts of Iran, Afghanistan and Pakistan. *Zataria Multiflora* essential oil (ZEO) is a hydrophobic concentrate with specific aroma present in the plant as secondary metabolite and due to its hydrophobic nature can facilely cross the membrane to reach inside the cell. Modern pharmacological studies indicate that EO extracted from *Z*. *Multiflora*, due its phenolic compounds possesses a wide range of biological properties including innate immune system stimulant^10^, antioxidant, antibacterial^11–13^ and antifungal activities^14, 15^. To the best of our knowledge, only a few reports have been published regarding the cytotoxic effects of ZEO. Previous studies have focused on the antiproliferative effect of EOs extracted from variety of plants and have elucidated that EOs execute their anticancer activity via multiple mechanisms involving apoptosis, cell cycle arrest, antimetastases, antiangiogenesis, increased levels of reactive oxygen and nitrogen species (ROS/RNS), DNA repair modulation, and etc. The effect of EOs and their constituents on tumor suppressor proteins (p53 and Akt), AP-1 and NF-κB transcription factors, MAPK-pathway, and detoxification enzymes like SOD, catalase, glutathione peroxidase, and glutathione reductase has also been reported^16^.

Previous studies have provided valuable data on the pharmacological properties of ZEO, but there are no substantial findings on the underlying cellular and molecular mechanism of ZEO cytotoxic properties on human Breast cancer cells. This study was carried out to assess the *in vitro* antiproliferative and cytotoxicity activities of ZEO in 2D and 3D cell cultures and its possible interaction with DNA.

## Results

### GC/MS analysis of ZEO

**Figure 1:**
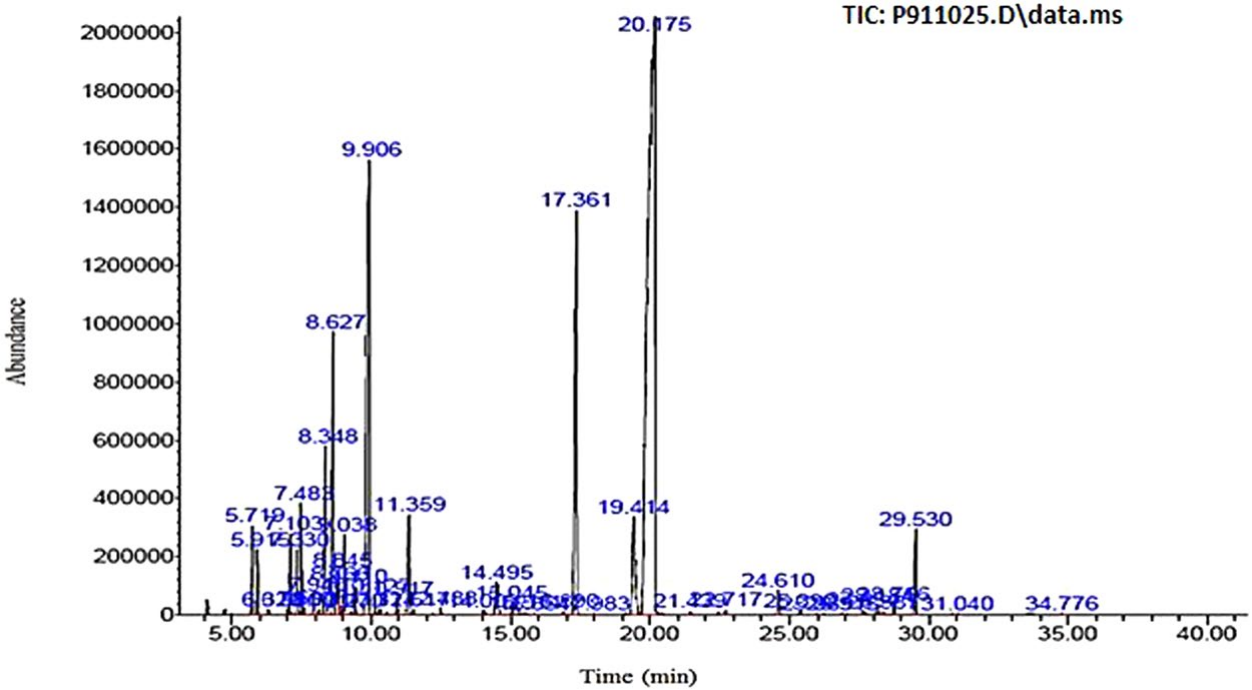
Gas chromatography-flame ionization detector (GC-FID) chromatogram of *Zataria Multiflora* essential oil. Elution time of peaks, component name and component percentage reported underneath. 5.719, α-Thujene (1.011%). 5.915, α-Pinene (0.765%). 6.320, Camphene (0.063%). 6.996, Sabinene (0.063%). 7.103, 1-Octen-3-ol (1.15%). 7.33, 3-Octanone(0.903%). 7.483, Myrcene (1.41%). 7.593, 3-Octanol (0.084%). 7.940, α-Phellandrene (0.27%). 8.131, δ-3-Carene (0.067%). 8.348, α-Terpinene (2.391%). 8.627, *p*-Cymene (4.299%). 8.766, Limonene (0.535%). 8.845, 1,8-Cineole (0.603%). 9.038, (Z)-β-Ocimene (1.085%). 9.270, Benzene acetaldehyde (0.051%). 9.41, (E)-β-Ocimene (0.414%). 9.906, γ-Terpinene (12.447%). 10.127, cis-Sabinene hydrate (0.24%). 10.324, trans-Linalool oxide (0.086%). 10.917, Terpinolene (0.268%). 11.359, Linalool (1.658%). 11.517, Hotrienol (0.084%). 12.488, allo-Ocimene (0.094%). 14.009, Borneol (0.098%). 14.495, Terpinene-4-ol (0.773%). 15.045, α-Terpineol (0.284%). 15.514, cis-dihydro carvone (0.05%). 15.854, trans-dihydro carvone (0.023%). 16.89, Thymol methyl ether (0.058%). 17.361, Carvacrol methyl ether (10.232%). 17.983, Unknown (0.016%). 19.414, Thymol (3.44%). 20.175, Carvacrol (52.275%). 21.439, Piperitenone (0.049%). 22.717, Carvacrol acetate (0.072%). 24.61, (E)-Caryophyllene (0.414%). 25.39, Aromadendrene (0.062%). 25.989, α-Humulene (0.02%). 26.915,Germacrene D (0.014%). 27.654, Viridiflorene (0.117%). 28.184, β-Bisabolene (0.164%). 28.387, γ-Cadinene (0.036%). 28.766, δ-Cadinene (0.186%). 29.53, cis-Sesquisabinene hydrate (1.514%). 31.04, Caryophyllene oxide (0.027%). 34.776, α-Bisabolol (0.036%).

It is well recognized that the medicinal properties of plants are largely attributed to the phytochemicals present in them. The oils isolated by hydrodistillation from the aerial parts of *Zataria Multiflora* were yellow liquids. The analysis of EO by GC/MS revealed 47 various compounds. Major compounds and their structures identified with respect to their specific peak values as represented in (Fig. 1 and Table 1). Carvacrol (52.2%), g-Terpinene (12.4%), Carvacrol methyl ether (10.23%), p-cymene (4.3%) and thymol (3.44%) were identified as ZEO’s major components.

**Table 1:** List of major compounds present in ZEO analyzed by GC-MS.

Compounds	Molecular formula	M.W.	RI	% of compound	structure
Carvacrol	C_10_H_14_O	150.217 g/mol	1300.479	52.275	
g-Terpinene	C_10_H_16_	136.24 g/mol	1058.4139	12.447	
Carvacrol methyl ether	C_11_H_16_O	164.24 g/mol	1243.4598	10.232	
p-Cymene	C_10_H_14_	134.21 g/mol	1023.073	4.299	
Thymol	C_10_H_14_O	150.217 g/mol	1291.845	3.44	

ZEO main components, their structures, Molecular Weights (M.W.) and Retention Indices (RI) are listed.

### MDA-MB-231 cell spheroids generation

**Figure 2:**
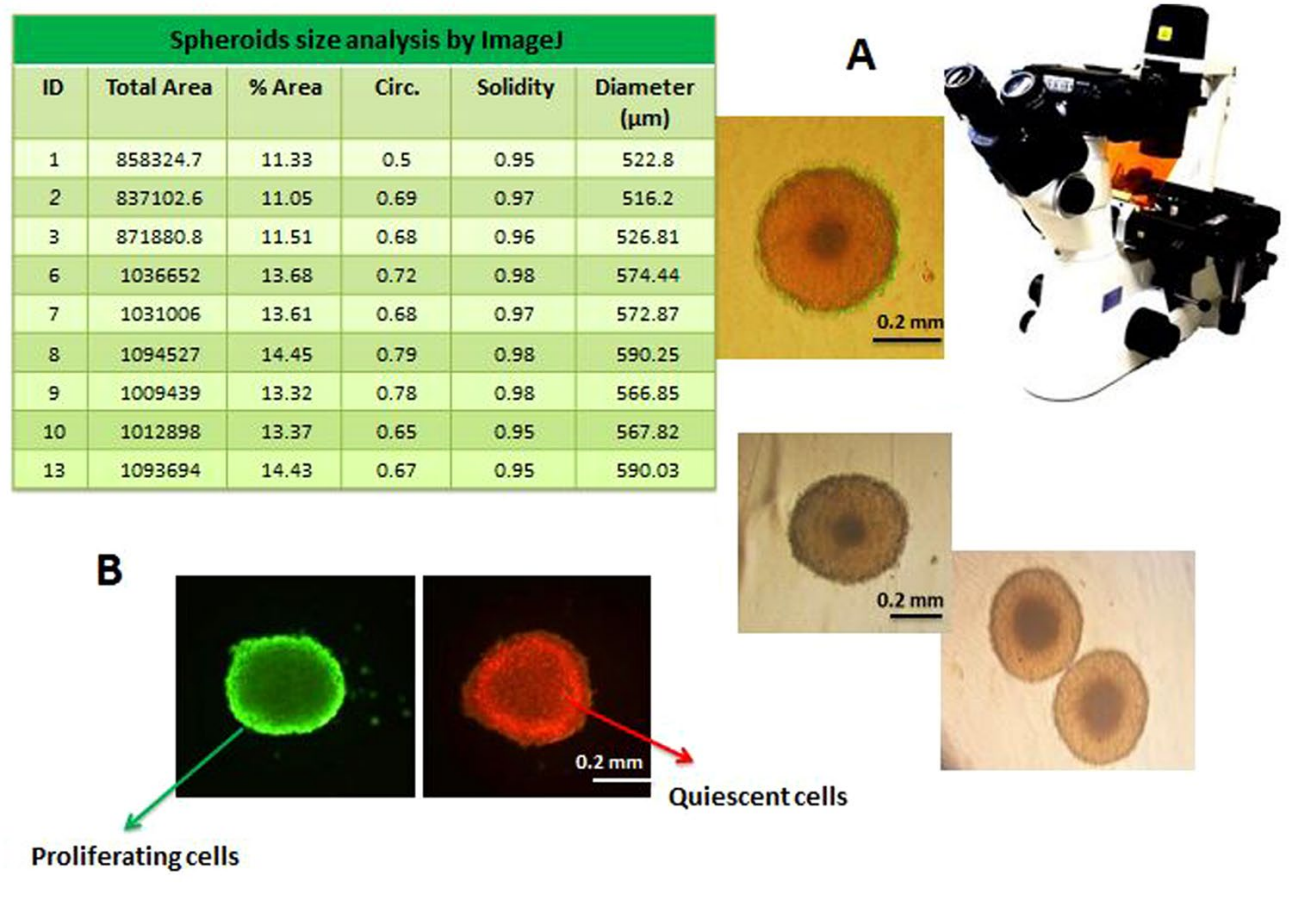
MDA-MB-231 cell spheroids. (**A**) MDA-MB-231 cell spheroid’s size and morphology inspected and imaged by bright field invert microscope. (**B**) AO and EB staining of a uniform spheroid, dark and orange highlighted regions (stained with EB) close to the center of the spheroid are mainly composed of quiescent/dead cells whereas, outermost layer which is highlighted in bright green (stained with AO) consists of proliferative, actively dividing cells.

Tumor cells are generally less sensitive to chemotherapeutics in solid tumor models such as cell spheroids than in monolayer cell cultures. Furthermore spheroid’s diameter is an important parameter in characterizing drug efficiency, for instance, small spheroids with diameters up to 200 μm are sufficient to reflect *in vivo*-like, cell-cell and cell-matrix interactions and are frequently used for anticancer drug screening^17^. However, spheroids with diameters of approximately 500 μm have been suggested to be a better choice than small spheroids, because they mimic the pathophysiological conditions of solid tumors, such as the specific hypoxic areas in the center and proliferation gradients more properly^18, 19^. For this reason, homogenous multicellular spheroids from MDA-MB-231 cells were generated by using Hanging Drop and subsequent maintenance and increase of spheroids size was achieved by using Liquid Overlay method^20^. In Hanging Drop method spheroids form under the effect of gravity as soft solid structures that can resist small physical impacts during handling. By using this method spheroids were generated in only 2 days and with subsequent combination with liquid overlay, after 4 days, ready to use cell spheroids with uniform size, shape, high cell density and minimum cell damage were generated. This is a simple, fast and low-cost method to generate multiple uniform spheroids with high viability. Dimensions of spheroids and viability of cells were controlled by the density of initial cells and time of culture. After 4 days, the growth of spheroids was monitored by measuring the volume and surface area of at least 100 spheroids by Image J software, and the average diameter of spheroids were calculated 555 ± 29.71 μm (Fig. 2A). Proliferation gradient of spheroids around 550 μm in diameter were also observable after this period: proliferative cells were located in the outer layer and quiescent cells were located near to the center where oxygen and glucose are scarce and cell death and necrosis often occurs(Fig. 2B)^19, 21^.

### ZEO induces cytotoxicity in Breast cancer cells monolayer (2D) and cell spheroids (3D)

**Figure 3:**
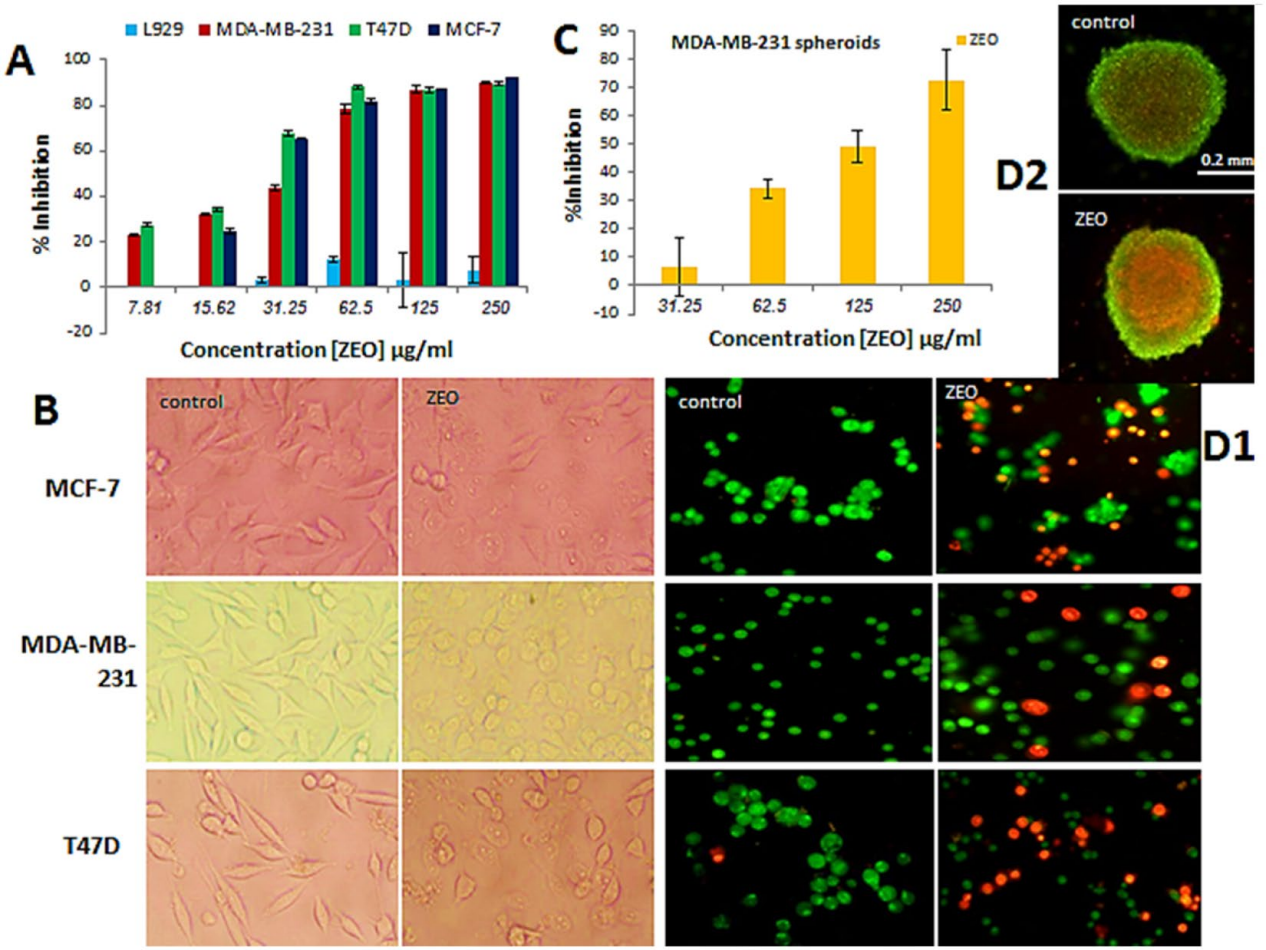
Inhibition of cellular viability by ZEO in monolayer and spheroid cell cultures. (**A**) L929, MCF-7, MDA-MB-231, and T47D cells were cultured in 96-well plates and treated with (7.81–250 μg/ml) concentrations of ZEO for 48 h and viability was determined by MTT assay. (**B**) Morphological changes of MCF-7, MDA-MB-231 and T47D Breast cancer cells after 24 h incubation with IC50 concentration of ZEO imaged by invert microscope. (**C**) MDA-MB-231 cell spheroids were exposed to 31.25–250 μg/ml concentrations of ZEO for 48 h and inhibition rate was determined by MTT assay. (**D1**,**D2**) AO/EB viability staining assay. MCF-7, MDA-MB-231, T47D 2D and MDA-MB-231 3D cell cultures after 24 h treatment with IC50 concentration of ZEO and staining with AO/EB showed clear apoptotic morphological changes in cells. Image of spheroid treated with ZEO indicates that it is not as compact as the control, suggesting spheroid integrity and size may have compromised by ZEO.

The cytotoxicity of ZEO on MCF-7 and T47D (luminal A, non-invasive, responsive to chemotherapy) and MDA-MB 231 (claudin-low, invasive, intermediate response to chemotherapy) cell lines^19^ which represent molecular subtypes of Breast cancers, along with normal fibroblast cell line L929 and MDA-MB-231 cell spheroids were evaluated after 48 h using MTT assay. As shown in (Fig. 3A,C), comparing the results of untreated (control) and treated groups, cells in monolayer culture and spheroids exhibited a dose-dependent decline in viability. The order of reduction in viability was as T47D > MCF-7 > MDA-MB-231 > MDA-MB-231 spheroids after 48 h of incubation and the IC50 values were found to be 20.09 > 25.06 > 29.89 > 118.4 μg/ml respectively. However, ZEO did not induce significant cytotoxic effect on L929 normal cells. These results indicate that ZEO has growth-inhibitory effect on Breast cancer cells while being non-toxic to normal fibroblast cells. Moreover The IC50 value of ZEO in approximately 500 μm diameter spheroids was nearly 4 times higher comparing to cancer cells monolayer. The differences in morphology of Breast cancer cells in the presence of IC50 concentration of ZEO were observed after 24 h by invert microscope (Fig. 3B). After treatment with ZEO, cell rounding, detachment, sometimes shrinkage and cytoplasmic vacuolation were clearly noticeable and supported the cytotoxicity results.

### ZEO induces apoptosis in MDA-MB-231 cells monolayer and cell spheroids

**Figure 4:**
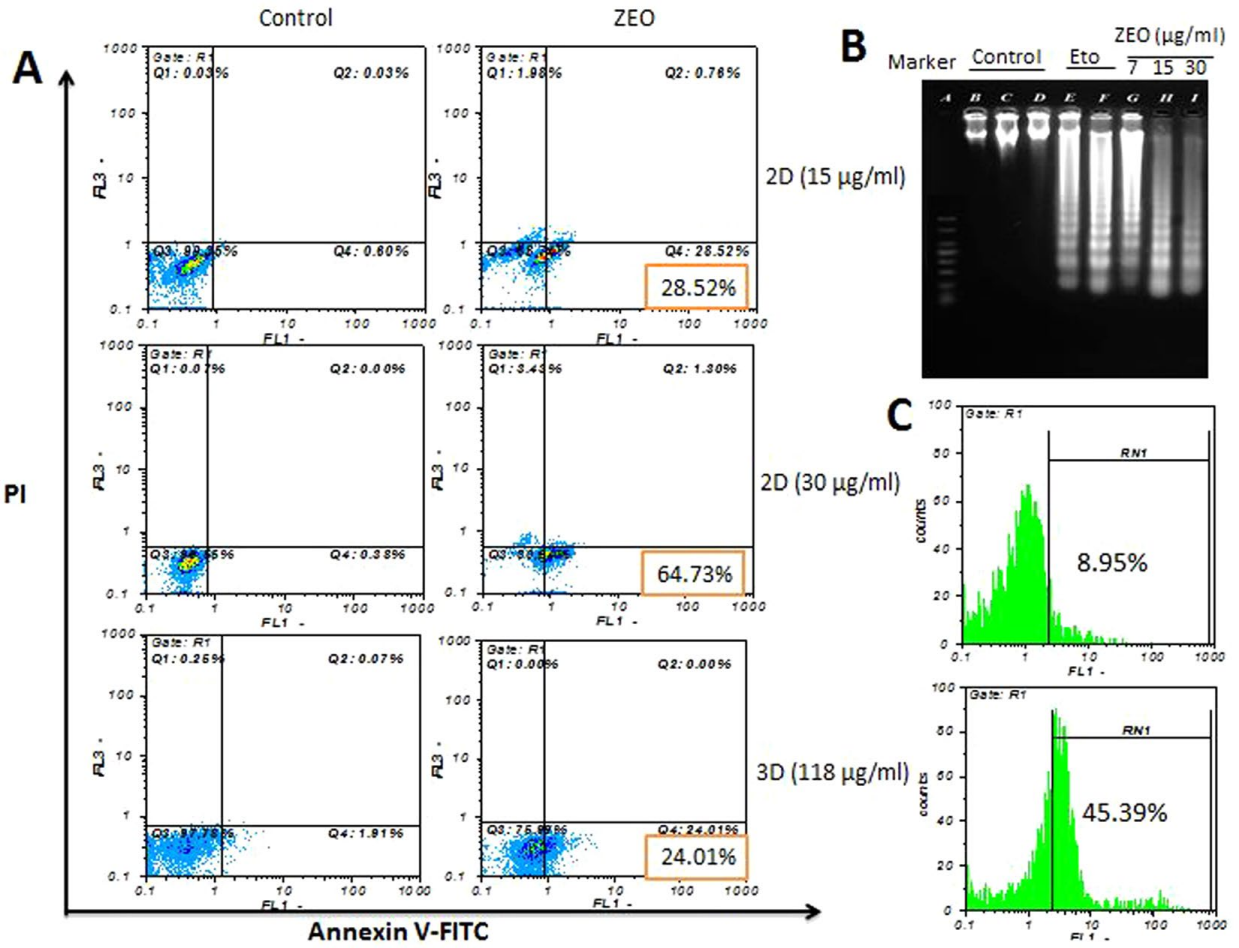
ZEO induces apoptosis in MDA-MB-231 cells 2D and 3D cell cultures. (**A**) Flow cytometry Scattergram of apoptosis and necrosis assay (Annexin V-FITC/PI) in MDA-MB-231 cells 2D and 3D cultures. In each panel, the lower left quadrant shows cells negative for both PI and Annexin V-FITC, upper left shows only PI necrotic cells, the lower right quadrant shows Annexin positive cells which are in the early apoptotic stage and the upper right shows both Annexin/PI positive, which are in the late apoptosis/necrosis stage. In all of the control samples more than 97% of the cells were live with intact cell membranes. While monolayer cultured samples treated with ZEO displayed considerable cytotoxicity and 27%(15 μg/ml), 64%(30 μg/ml) of their cells were in early apoptotic phase, 76% of the cells in 3D tumor spheroids treated with ZEO were alive with intact cell membrane and only 22% of the cells were in early apoptotic stage. (**B**) DNA fragmentation analysis on agarose gel. DNA was isolated from monolayer cultured MDA-MB-231 cells, treated for 7 h with 7, 15 and 30 μg/ml of ZEO and etoposide (positive control), electrophoresis was performed in 2% agarose gel, followed by Ethidium bromide staining. (**C**) TUNEL assay flow cytometric histograms of MDA-MB-231 cells treated with ZEO IC50 for 4 h and untreated control. An evident shift of the cell population (45.39%) to the right compared to control (8.95%) indicates a significant apoptotic cell population in ZEO treated cells.

As viability assay revealed, ZEO potently inhibits cancer cells growth both in monolayer and spheroid cell cultures. It also induced distinguished morphological changes in the treated cells which strongly suggest that ZEO may have caused apoptosis in cancer cells. In order to verify ZEO induced apoptosis: AO/EB staining, AnnexinV-FITC/PI staining, TUNEL assay and DNA fragmentation assay was performed. In AO/EB staining of MCF-7, MDA-MB-231 and T47D cells in monolayer cell culture, cell nuclei and cell membrane integrity of control group did not changed significantly, while the experimental groups revealed different extents of chromatin condensation, nuclear fragmentation and destruction of cell membrane integrity. As it is shown in (Fig. 3D1,D2) the same staining was executed on control and treated MDA-MB-231 cell spheroids on day 4. It is worth noting that in comparison to monolayer cell culture, cell viability has been less compromised in cell spheroids. Flow cytometry analysis was performed on MDA-MB-231 cell monolayer and spheroids, using the Annexin V-FITC and propidium iodide (PI) double staining after being incubated with 15 μg/ml ZEO (1/2 IC50) and 30 μg/ml (IC50) concentrations in monolayer for 4 h and spheroids (IC50) for 24 h. As shown in (Fig. 4A) ZEO markedly induced early apoptosis in MDA-MB-231 cells in a concentration dependent manner. Specifically after treatment with 15 μg/ml of ZEO for 4 h the early apoptotic cells population was 27.92%, higher than the control group, while after treatment with 30 μg/ml for 4 h, it significantly increased and reached 64.35%. ZEO also induced apoptosis in MDA-MB-231 cell spheroids (Fig. 4A). After treatment of spheroids for 24 h with 118 μg/ml (IC50), the percentage of early apoptotic cells were 22.1%, higher compared to control group. In addition to apoptosis specific morphological changes, a representative hallmark of apoptosis is DNA Degradation into oligonucleosome-size fragments in a distinctive event in apoptosis^22^. In the present study, this fragmentation event was observed qualitatively by resolving cellular DNA on agarose gel and quantitatively by TUNEL assay. In TUNEL assay, after treatment of MDA-MB-231 cells with IC50 concentration of ZEO for 4 h, DNA fragmentation was quantified by flow cytometry and Compared to untreated cells up to 45.39% apoptosis was observed (Fig. 4C). DNA fragmentation analysis on agarose gel which is the gold standard to confirm the induction of apoptosis also showed the apoptosis characteristic ladder pattern in MDA-MB-231 cells. It is worth to specify that DNA laddering pattern was more evident in three doses 7, 15 and 30 μg/ml (Fig. 4B).

### ZEO induces intracellular ROS generation, loss of ΔΨm and increases caspase-3 activity in MDA-MB-231 cells

**Figure 5:**
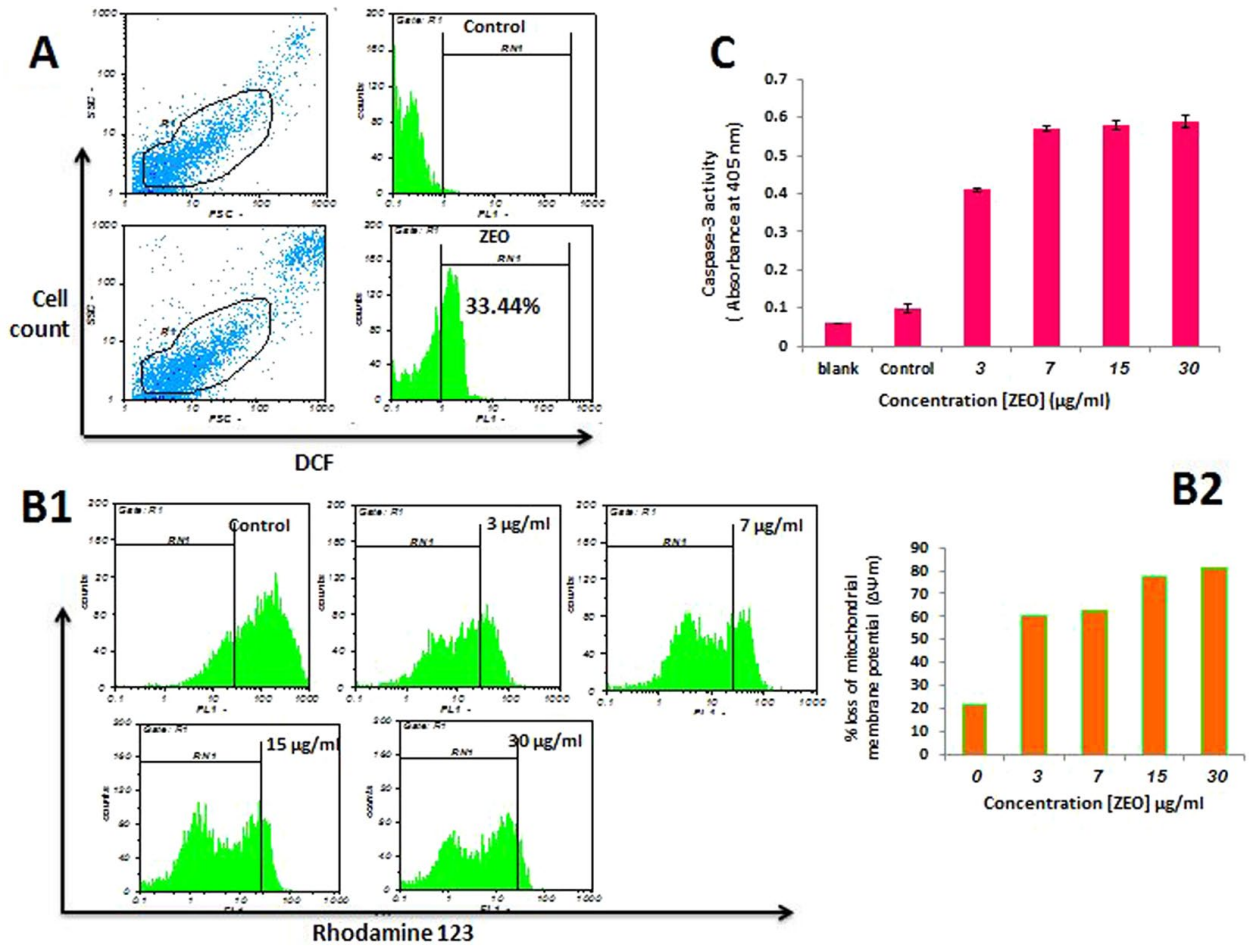
ZEO induces ROS production in MDA-MB-231 cells. (**A**) ROS level in MDA-MB-231 cells were estimated after incubation with IC50 concentration of ZEO using DCFH-DA dye and flow cytometer. (**B1**,**B2**) The collapse of mitochondrial membrane potential. The ΔΨm of MDA-MB-231 cells reduces increasingly after incubation with 0–30 μg/ml ZEO for 12 h, as assessed by Rhodamine123 staining and FACS analysis. (**C**) caspase-3 Activation assessment after 4 h treatment with ZEO (3–30 μg/ml).

There is increasing evidence that ROS induced by apoptotic stimuli leads to mitochondrial dysfunction and we hypothesized that ZEO may have caused apoptosis in MDA-MB-231 cells via increasing ROS production. It has been shown that ZEO usually causes oxidative stress therefore increase of ROS levels in MDA-MB-231 cells was measured by the ROS-specific fluorescent dye (DCFH-DA). The ROS-specific fluorescent staining assay confirmed that ROS level rapidly increases in MDA-MB-231 cells treated with IC50 concentration of ZEO after 12 h (34.44%) (Fig. 5A). ROS augmentation may oxidize mitochondrial pores and therefore disturb MMP (mitochondrial membrane potential) and cause apoptosis. MMP is a major event in mitochondrial apoptotic pathway^23^. Rhodamine 123, a fluorescent dye with the ability to be selectively absorbed by mitochondria, was used to evaluate the effect of different concentrations of ZEO on MMP in MDA-MB-231 cells. Following ROS damage to the cell, the membrane permeability of mitochondria increases and the MMP decreases^24^. A significant reduction of MMP in a dose- dependent manner after treatment with 3–30 μg/ml of ZEO was observed which indicates that the mitochondrial apoptosis pathway may have involved in apoptosis-induced by ZEO (Fig. 5B1,B2). Caspase-3, a key effector in the process of apoptotic cell death^24^ is a cysteine proteases which mediates apoptosis by proteolysis of specific substrates, that are considered the primary executioners of apoptosis^25^. To analyze whether ZEO triggers apoptosis in a caspase dependent manner, the activity of caspase-3 was measured. Caspase-3 activation capability in MDA-MB-231 cells was evaluated after incubation of cells with 3, 7, 15 and 30 μg/ml of ZEO for 4 h. The activity of caspase3 increased nearly 4.3, 5.6, 6.15 and 6.2 times respectively compared to the negative control (Fig. 5C).

### ZEO treatment leads to DNA damage (DNA oxidation and strand break) in MDA-MB-231 cell

**Figure 6:**
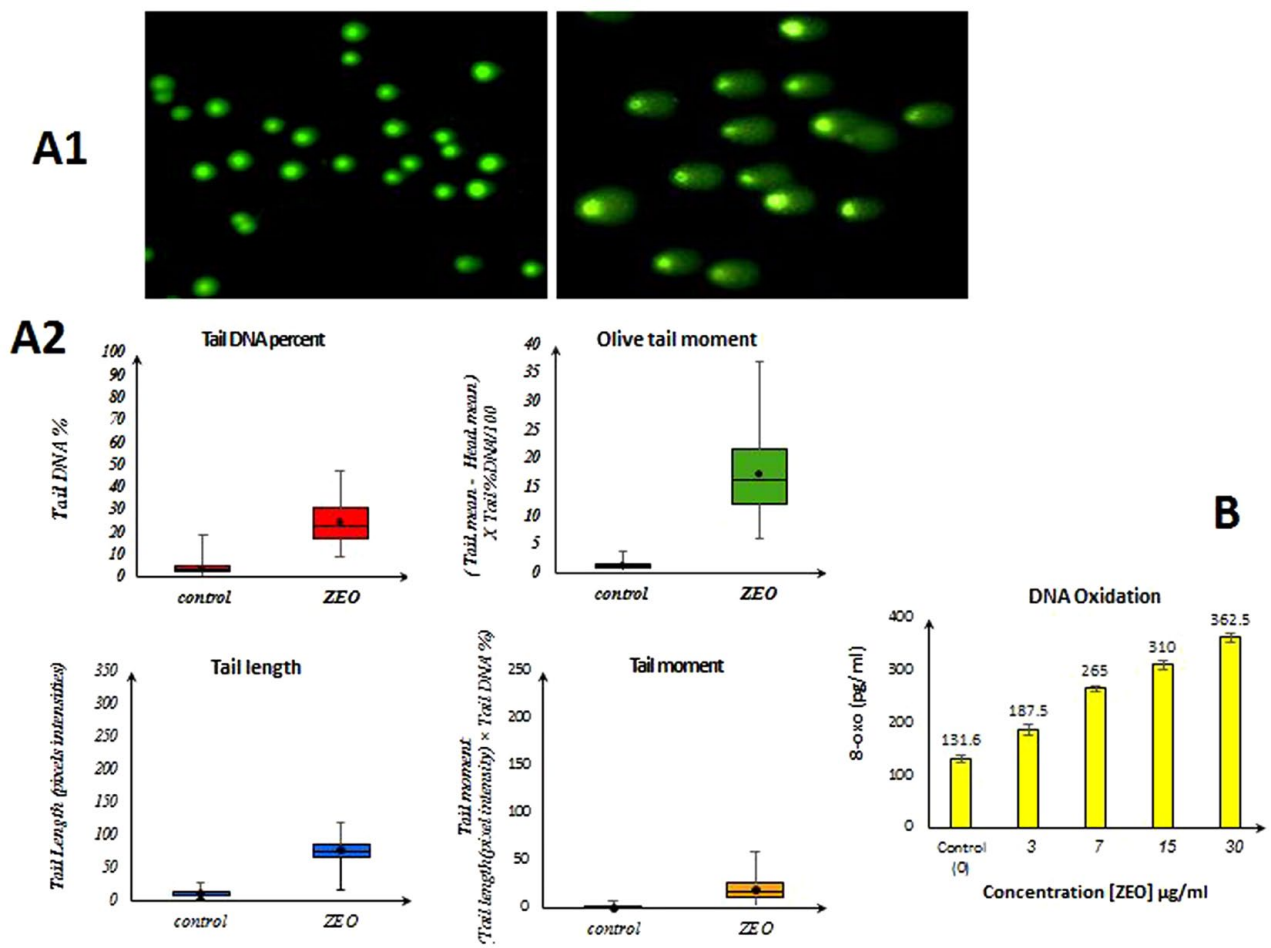
ZEO induces DNA damage in MDA-MB-231 cells. (**A1**) Images of samples treated with IC50 concentration of ZEO, untreated controls and parameters measurement by Open Comet software. (**A2**) DNA strand break results in single cells after 2 h incubation with IC50 concentration of ZEO in MDA-MB-231 cells. The comet assay results were obtained by analyzing tail DNA percentage, tail moment, tail length and olive tail moment are presented in box and whiskers plot. (**B**) MDA-MB-231 Genomic DNA oxidation produces increasing amounts of 8-oxo upon incubation with ZEO (3–30 µg/ml) compared to untreated control.

Increased levels of ROS in ZEO treated cells may cause DNA damage. Hence to investigate the effect of ZEO on DNA oxidation, production of 8-oxoguanine (8-oxo) as the most common oxidative base lesion was evaluated^26^. As shown in (Fig. 6B), exposing MDA-MB-231 cells for 2 h to increasing concentrations of ZEO (3, 7, 15, 30 µg/ml) has resulted in increased formation of 8-oxo (131.6, 187.5, 265, 310, 362.5 pg/ml) in their Genomic DNA. To further confirm the induction of DNA damage by ZEO in MDA-MB-231 cells, after 2 h incubation with IC50 concentration, the level of nuclear DNA integrity was determined by comet assay under alkaline electrophoresis condition. The degree of DNA migration in MDA-MB-231 cells was increased after treatment by ZEO (Fig. 6A1). These results indicate that the apoptosis induced by ZEO is tightly correlated with DNA oxidation and strand break. The comet assay data analysis represented as tail DNA percentage, tail length, tail moment and Olive tail moment in the corresponding histograms (Fig. 6A2).

### ZEO induces G1 and G2/M phase cell cycle arrest in monolayer and S phase arrest in spheroids

**Figure 7:**
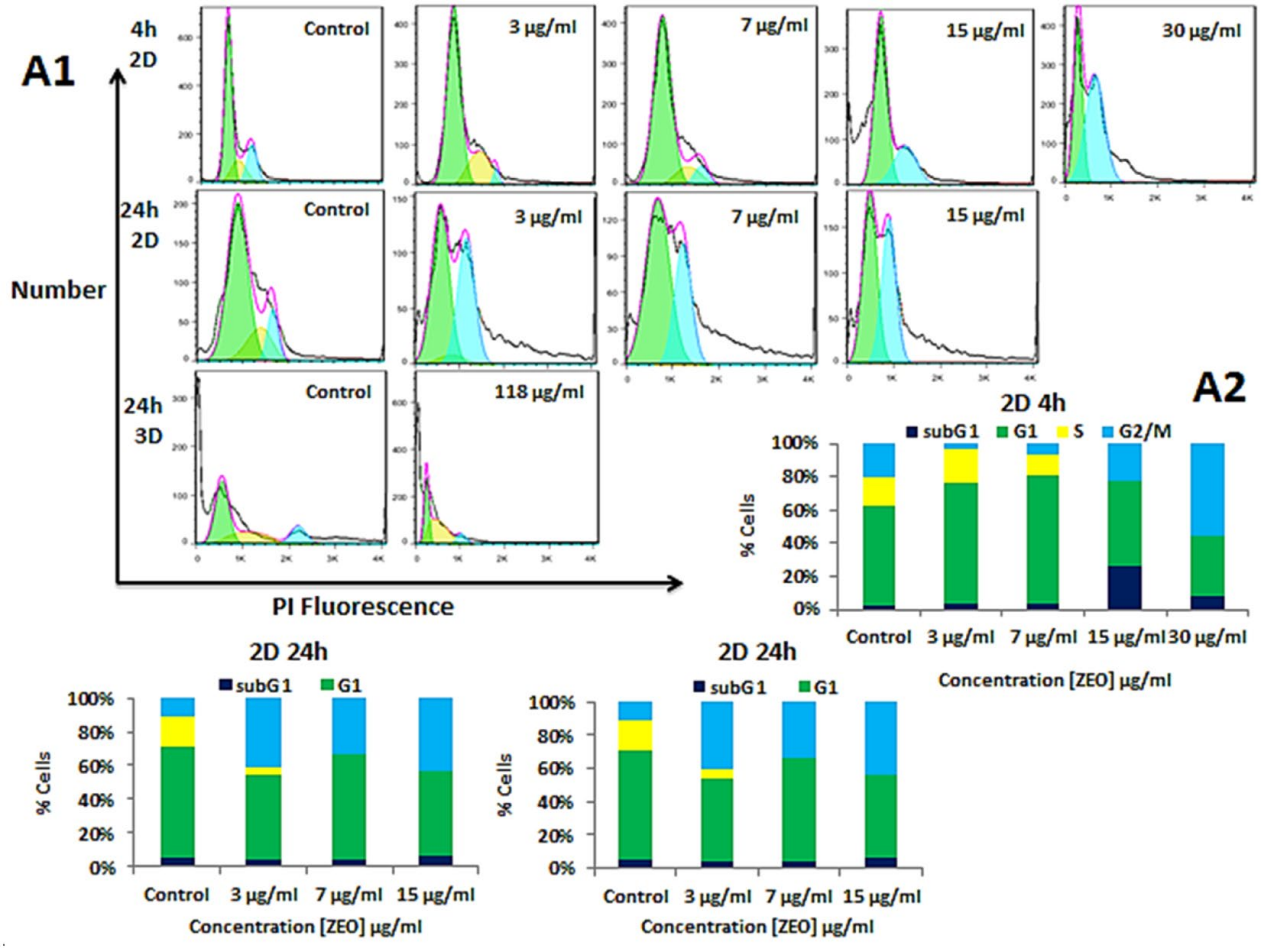
Effect of ZEO on DNA content in 2D and 3D cell cultures. (**A1**) DNA profile of MDA-MB-231 cells in monolayer treated with different concentration of ZEO for 4 and 24 h, and MDA-MB-231 cell spheroids incubated with IC50 concentration of ZEO for 24 h using flow cytometry. (**A2**) Percentage of cells population in sub-G1, G1, S and G2/M phases in 2D and 3D cell cultures analyzed with flowJo software.

It is reported that some anticancer agents whether directly induce apoptosis or arrest the cells at G0/G1, S and G2/M phases of the cell cycle and then induce apoptosis to kill cancer cells. The effect of ZEO on cell cycle distribution of monolayer MDA-MB-231 cells revealed that ZEO treatment arrests cells in G1 and G2/M phases in a dose and time-dependent manner and also induces apoptosis as confirmed by increase in the sub-G1 population. After 4 h incubation with 3 and 7 μg/ml of ZEO, accumulation of cells in G1 phase was observed while at 15 and 30 μg/ml concentrations cells exhibited G2/M phase arrest (Fig. 7). Additionally, our results indicated that after 24 h treatment by 3, 7 and 15 μg/ml concentrations of ZEO, cells arrest 30%, 25%, and 34% in G2/M phase of cell cycle respectively. While only 10% of untreated controls were in G2/M phase (Fig. 7). On the other hand, the treatment of MDA-MB-231 cell spheroids for 24 h with IC50 concentration of ZEO resulted in a marked accumulation of cells in the sub-G1 phase, suggesting a significant apoptotic, but not necrotic, cell death. It is worth notifying that, the ZEO induced S phase cell cycle arrest in MDA-MB-231 cell spheroids is intrinsically different in nature compare to 2D monolayer (Fig. 7).

### UV-Visible, fluorescence and circular dichroism spectroscopy shows ZEO interacts with DNA via intercalation

**Figure 8:**
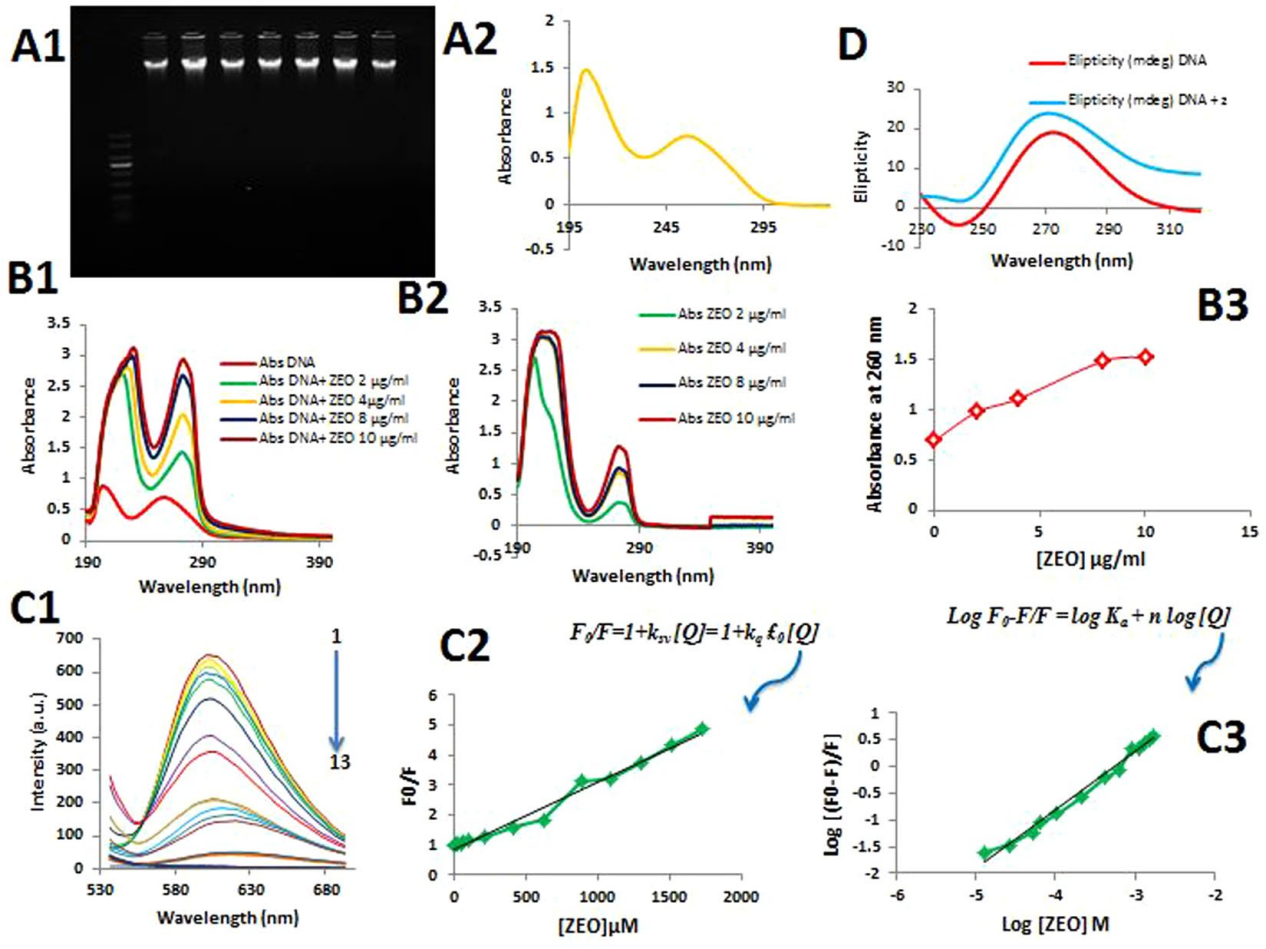
(**A1**,**A2**) High molecular weight DNA on 0.8% agarose gel after 40 min electrophoresis at 5 v/cm and UV–Visible spectra of rat hepatocyte genomic DNA. (**B1**,**2**,**3**) UV absorption spectra of a: 0–32 μg/ml of ZEO, b: DNA with various concentrations Of ZEO c: DNA/ZEO absorption at 260 nm (**C1**) Fluorescence quenching spectra of EB–DNA solutions (C_EB_ = 2.6 μM, C_DNA_ = 50 μg/ml) upon addition of increasing amounts of ZEO (2–224 μg/ml)  (pH = 7.4, T = 310 K, ex = 500 nm, em = 530–700 nm). (**C2**,**C3**) Stern–Volmer plots for the mechanism fluorescence quenching of EB–DNA by ZEO (**D**) Circular dichroism spectra of the rat hepatocyte genomicDNA (100 μg/ml) and DNA/ZEO (16 μg/ml) at 298 K.

DNA is the primary intracellular target for a wide range of anticancer agents and DNA-drug interaction determines the cells fate by altering transcription and/or replication inhibition^27–29^. After DNA extraction, its concentration and quality was evaluated. Samples with absorbance ratio (A260/A280) ≥1.8 were accepted as pure genomic DNA. Due to chromophoric groups in purine (adenine and guanine) and pyrimidine (cytosine and thymine) moieties responsible for the electronic transitions DNA molecule has a maximum absorption at 260 nm^28^. To verify extracted DNA integrity electrophoresis was perform on 0.8% agarose gel to ensure high molecular weight DNA (Fig. 8A1,A2).

UV–Vis absorption spectroscopy is an effective, simple and most common method to investigate the interaction of small molecules with genomic DNA^28, 30^. In this method in order to establish whether there is any interaction between DNA and the drug, DNA spectrum intensity modifications and maximum absorption shifting are inspected from when the ligand is free in solution to when the ligand is bound to DNA. As shown in (Fig. 7B1–B3), ZEO maximum absorption bands were at 200–220 and 280 nm, by adding increasing amounts of ZEO (0–32 μg/ml) into constant amount of DNA solution (50 μg/ml), DNA absorption intensity at 260 nm obviously increased accompanied by a significant 16 nm red shift (276 nm). This magnitude of shift in the absorption peak position is correlated with the strength of interaction^30^. Adding increasing amounts of ZEO to DNA solution caused hyperchromicity and red shift phenomena which indicate the intercalation of ZEO molecules between two strands of DNA^29, 31^.

Fluorescence spectroscopy is a useful technique to study the interaction of a chromophore with other molecules. Since DNA molecule per se is not a chromophore and no fluorescence was observed for the ZEO. Therefore, Ethidium Bromide (EB) was added to DNA solution as an intercalative probe and enhanced DNA fluorescence dramatically at 600 nm^31^. Hence, whenever a second ligand, which competes for the DNA binding sites, is added, it substitutes the EB and fluorescence quenching occurs. The emission spectra of the EB–DNA solutions in the presence of the increasing amounts of ZEO have shown in Fig. 7C which clearly exhibits a decrease in the fluorescence intensity at 600 nm. Quenching mechanism and binding parameters in competitive binding experiment at the presence of ZEO and EB was identified according to Stern-Volmer equation as described previously^32, 33^. As it is illustrated in Fig. 7C b&c Stern-Volmer plot is linear and binding parameters are n = 1.1 and K_a_ = 0.37 × 10^4^ which indicate that quenching mechanism is dynamic and binding mode is intercalation respectively.

Circular dichroism (CD) is a technique to assess conformational changes, binding mode and interaction affinity of small molecules with biomolecules. Inspecting modifications of CD signals at 200–320 nm is informative to detect and follow DNA conformational changes, damage and/or cleavage upon interaction with drugs^34^. Rat hepatocyte genomicDNA displayed the typical spectrum of B-form DNA with positive band at 275 nm and a negative band at 245 nm due to base stacking and right-handed helicity respectively^35^. The CD spectra of DNA in the presence of the ZEO are shown in (Fig. 7D). In the presence of ZEO, DNA CD spectra positive and negative peak intensities increased which indicates the stabilization of the right-handed BDNA.

## Discussion

Despite all advances in Breast cancer treatment, due to limited efficacy of chemotherapeutics the disease incidence is growing and efforts to develop novel and effective treatments has been fruitless^4^. Currently, natural compounds and herbal medicines in particular, has become more popular than synthetic drugs and provided an alternative treatment option for patients. Relentless proliferation of cancer cells, place the drugs with the ability of inhibiting DNA synthesis among the best choices to treat cancer^36^. A wide range of anticancer drugs aim DNA as the primary intracellular target and regulate cell functions by modulating transcription or replication inhibition in a way which leads to programmed death of cancer cells^37–39^. The anti-proliferative effect of ZEO has been previously reported in cancer cells^40^, however underlying cellular and molecular mechanism for antitumor properties of ZEO has not been elucidated. Therefore in the present study, we initially have evaluated the antiproliferative and anti-apoptotic effects of ZEO, a polyphenolic Essential Oil isolated from *Zataria Multiflora* in 2D and 3D cell cultures, followed by its possible apoptosis mechanism in MDA-MB-231 cells and Interaction properties with DNA.

According to GC/MS analysis, the major components in hydrodistilled ZEO are oxygenated monoterpenes, monoterpene and sesquiterpene hydrocarbons, oxygenated sesquiterpene and a non-phenolic portion^41^. Although the essential oil’s main components remain the same between plants from different geographical regions, but their relative quantities differ in various regions. Some reported carvacrol as the dominant compound, while others have reported thymol “an isomer of carvacrol” as the main constituent of the fresh plant. However carvacrol is the primary constituent in the dried plant. It is clear that geographical variation, cultivar differences, stage of plant growth, preparation process and other factors may influence oil composition both quantitatively and qualitatively^15^. According to our findings, the essential oil of *Zataria Multiflora* contains substantial amounts of carvacrol, which is renowned for its anti-oxidant, anti-microbial and anti-fungal properties and also has been reported to possess cytotoxic effects on cancer cells *in vitro* and *in vivo* ^42, 43^.

One of the goals of this study was to validate the cytotoxicity effect of ZEO on Breast cancer cells specifically in multicellular spheroids of MDA-MB-231. Traditionally, *ex vivo* drug screening is performed on conventional 2D monolayer cell cultures. However 3D cultures such as cell spheroids may be more physiologically relevant. Cells growth in multicellular spheroids similar to what observed in tumors *in vivo* have lower proliferation rate compared to 2D cell cultures. As such, it is reported that multicellular spheroids are superior models for preclinical drug screening. Multiple methods have been implemented so far to generate tumor spheroids *in vitro*, however these have not been largely applied, as they require specialized equipment, and complicated protocols^21, 44^. In this study we have generated spheroids with high size and volume uniformity and high circularity. 5000 cells per spheroid were produced in short culture time (4 days) by combination of liquid overlay and hanging drop methods. ZEO exhibited inhibition against the examined cancer cells (MDA-MB-231, T47D and MCF-7) in a dose-dependent manner and was significantly less toxic to normal fibroblast cells (L929), indicating a favorable selectivity toward cancer cells. The cytotoxic property of ZEO may be due to the presence of phytochemicals such as polyphenolic compounds in the EO. However, our results indicate that spheroids were more resistant to the toxic effects of the ZEO compared to 2D cultures.

The induction of apoptosis is one of the principle mechanisms by which anti-cancer drugs kill cancer cells. MDA-MB-231 cells treated with ZEO exhibited certain apoptotic cell characteristics such as cell rounding, cell shrinkage and cytoplasmic condensation, as well as presence of sub-G1 phase cell population. Externalization of membrane phosphatidylserine is an indication of programmed cell death which is quite detectable by increased binding of FITC conjugated Annexin V, a calcium dependent phospholipid binding protein. The number of early apoptotic cells increased in a concentration-dependent manner in MDA-MB-21 cells, treated with ZEO. A hallmark of apoptosis is the fragmentation of nuclear DNA that was clearly evident in the cells treated with ZEO. Mitochondria play a crucial role in the apoptosis induced by synthetic and natural anti-cancer agents and many of these agents implement their antitumor activity through ROS-dependent apoptotic signaling. ROS are by products of cellular respiration and play a crucial role in apoptosis^45^. Elevated cellular ROS levels leads to the loss of mitochondrial membrane potential, which causes the release of pro-apoptotic factors and the activation of caspase-3. Caspase-3 activation subsequently leads to DNA breakage, nuclear chromatin condensation, and apoptosis^25, 46^. Treatment of MDA-MB-231 cells with ZEO resulted in the mitochondrial dysfunction, accompanied with ROS augmentation and ROS level considerably increased after cells were exposed to ZEO, ZEO-induced loss of mitochondrial membrane potential, activity of Caspase-3 enhanced by different concentration of ZEO, resulted in oxidative stress and free radicals attack to DNA, leaving DNA oxidation and single and double strand breaks, which causes apoptosis^47^.

Uncontrolled cell proliferation is the typical characteristic of cancer and cell cycle arrest another major target for cancer therapy, similarly can contribute to the inhibition of cancer cells proliferation. Cell cycle distribution status is often considered as a key parameter in cell survival, growth and proliferation. Our results indicate that ZEO treatment of monolayer MDA-MB-231 cells can induce G1 and G2/M cell cycle arrest, reducing the number of cells in S phase and induce apoptosis. Whereas ZEO treated spheroids showed S phase arrest in MDA-MB-231 cell spheroids followed by increased accumulation of cells in Sub G1 phase. These data together demonstrated ZEO induces cell cycle arrest in 2D and 3D cell cultures at completely different phases, this is because cells in 3D had a rounder morphology and a wider array of cell-cell and cell-ECM interactions and mimic tumor physiological environment more realistically. Since DNA replication is an essential phase of the cell cycle, many of the cytotoxic agents induce their effect via covalent or noncovalent interaction with DNA. Hence ZEO and rat hepatocyte genomic DNA interaction was explored via spectroscopy. Alterations in DNA UV-Vis spectra at 260 nm such as Hyperchromicity and red shift by adding increasing amounts of ZEO indicated that ZEO has induced conformational changes in DNA by forming DNA-ZEO complexes which causes deformation and instability in DNA structure. ZEO in competitive fluorescence substituted ethidium bromide this shows that ZEO can intercalate in DNA. Additionally observing hyperchromicity at 275 nm positive band and 245 nm negative band of CD spectra also confirmed ZEO intercalates in DNA.

In summary, our findings indicate that ZEO by inducing apoptosis via increasing intracellular ROS level and direct genome targeting executes its antiproliferative effect on MDA-MB-231 cells in both 2D and 3D cell cultures without considerable cytotoxicity against normal fibroblast cells. Furthermore, MDA-MB-231 cell spheroids in 3D cell culture demonstrated more resistance to ZEO cytotoxic effects Compared to 2D monolayer cell culture.

## Methods

### Plant collection and extract preparation

The leaves and flowers of *Zataria Multiflora* (Lamiacea) were collected in May month from mountainous areas of Shiraz in Iran. Consequently ZEO was extracted from the air-dried through hydrodistillation using the all-glass Clevenger-type apparatus. The density of ZEO calculated by digital balance (Acculab, Sartorius group, Germany) and the average was reported 996 mg/mL. Therefore, each µL of ZEO approximately equals to 1 mg.

### Essential oil analysis

GC/MS analyses were executed on Agilent 7890 A gas chromatograph equipped with column HP-5 (30 m length, 0.25 mm i.d., film thickness 0.25 μm) coupled with FID detector. Carrier gas was He with split ratio of 1: 40. Temperature was programmed at 60 °C rising to 240 °C, Injector temperature 240 °C, detector temperature 250 °C and injector volume was 0.1 μl. GC-MS was performed using Agilent 5975C coupled with Agilent 5975C mass spectrometer equipped with a column HP-5MS (see GC), using the same temperature programed and carrier gas as above and ionization source temperature of 280 °C.The constituents of the essential oils were identified by calculation of their retention indices and comparison of their mass spectra with those of the internal reference mass spectra library.

### Cell lines and culture

MDA.MB.231 (highly invasive human breast cancer cell line), MCF-7 (human breast adenocarcinoma cell line), T47D (human breast cancer epithelial cell line) and L929 (normal fibroblast cell line) were purchased from National Cell Bank of Iran (Pasteur Institute, Iran). All the cells were grown in RPMI-1640 medium (Gibco) with 10% FBS (Gibco) supplemented with antibiotics (100 U/ml penicillin and 100 μg/ml streptomycin). Cells were maintained at 37 °C under humidified air containing 5% CO2 and were passaged using trypsin/EDTA (Gibco) and phosphate- buffered saline (PBS) solution. Culturing media and conditions used to grow the cells as 3D colonies was the same as monolayer cell culture.

### Spheroid formation using hanging drop and liquid overlay methods

Generation of spheroids was carried out using combination of liquid overlay and hanging drop cultivation techniques. singled cell suspension of MDA-MB-231 at the concentration of 1.0 × 10^6^ cells/10 ml (for 5000 cells per spheroid) in complete culture medium was deposited in 50 μl drops on the inner side of a petri dish lid then petri dish lid turned upside down and was placed on top of the plate filled with 10 ml sterilized water (to humidify the culture chamber after distribution of the drops). Cells under the force of gravity will be aggregated at the bottom of the hanging drop. Small spheroids were formed on day 3 (after 2 days) and were collected by widened end tips (100 μl pipette tip cut widely 5–6 mm from the end). After the formation of the spheroids, to increase the size of spheroids, they were transferred into 96-well plates, precoated with 50 μl of 0.5% poly-HEMA (sigma) in 95% ethanol and air dried at 37 °C for 3 days prior to use. Then homogenous sized spheroids were added to each well of a round bottom poly-HEMA–coated 96-well plate and supplied with 200 µl fresh medium. The plates were incubated under standard cell culture conditions at 37 °C, 5% CO_2_ in humidified incubator.

### Measurement of spheroid size and Image Analysis

Cell spheroids with diameters ranging from 500–600 μm were obtained in about 4 days, the morphology and integrity of MDA-MB-231 cell spheroids were imaged at 5 × and 10× magnitudes using invert microscope (Ax overt 25, Zeiss, Germany). Volume and size of spheroids were analyzed with Image J software.

### MTT cell viability assay in MDA-MB-231 Multicellular spheroids and MDA-MB-231, T47D, MCF-7, L929 Monolayer Cells

Cell growth and cell viability were quantified using the MTT [3-(4, 5-dimethylthiazol-2-yl)-2, 5-diphenyltetrazolium Bromide] (Sigma-Aldrich) assay. In brief, for monolayer culture, All the cells (MDA-MB-231, MCF-7, T47D and L929) were digested with trypsin, harvested, adjusted to a density of 1.4 × 10^4^ cells/well and seeded to 96-well plates filled with 200 μl fresh medium per well for 24 h. When cells formed a monolayer, treated with 0–250 μg/ml ZEO for 48 h at 37 °C in 5% CO2. MTT assay for spheroids were carried out with slight modifications as will be described. At the end of the treatment (48 h), while the monolayer culture was left untouched in the original plate, spheroids of each well were transferred to a flat bottom 96-well plate, afterwards supernatant was removed and 200 μl/well of MTT solution (0.5 mg/ml in phosphate-buffered saline [PBS]) was added and the plate was incubated at 37 °C for an additional 4 h. MTT (the supernatant of cells were) was removed and dimethyl sulfoxide was added (100 μl per well). Cells were incubated on a shaker at 37 °C until crystals were completely dissolved. Cell viability were quantified by measuring absorbance at 492 nm using a ELISA reader (Model wave xs2, BioTek, USA). The concentration of ZEO that resulted in 50% of cell death (IC50) was determined from respective dose-response curves.

### Fluorescent Staining in monolayer and spheroids

Fluorescent staining ethidium bromide (EB)/acridine orange (AO) (Sigma-Aldrich) was performed to assess rates of cellular viability (Live/Dead). Initially cells were seeded in 6 well (monolayer) and 24 well (spheroids) cell culture plates and treated with IC50 concentration of ZEO for 24 h. then cells and spheroids washed with PBS, after which, a solution containing EB/AO was added, the stained cells were immediately visualized and imaged under an fluorescence microscope (Axioskop 2 plus, Ziess, Germany). The differentiation between viable, apoptotic, and necrotic cells is based on the difference between dye permeability into intact cell membrane. Green cells represent viable cells and are stained only with AO, green and orange cells with condensed chromatin represent early and late apoptotic cells and are stained with both AO and EB (with moderate alteration in membrane permeability), finally necrotic cells are orang and stained with EB. Ten photos were taken of randomly selected areas of the stained slides to ensure that the data obtained were representative.

### Detection of apoptosis by Annexin V-FITC and PI staining in monolayer and MDA-MB-231 cell spheroids

Identification of apoptotic and necrotic MDA-MB-231 cells treated with ZEO was performed using the Annexin V-FITC apoptosis kit (BioVision). After treating cells for 4 h with the IC50 concentration of ZEO, cells were harvested, thoroughly washed and labeled with PI and FITC according to the protocol described by the kit manufacturer. The distribution of differentially labeled cells was identified by flow cytometry (Partec PAS, Germany).

### TUNEL Assay for *In Situ* Apoptosis Detection

Terminal deoxyribonucleotidyl transferase-mediated dUTP nick-end labeling (TUNEL) *in situ* apoptosis assay was used to detect DNA fragmentation using *In situ* cell death detection kit, fluorescein (Roche) according to the manufacturer’s instruction. Briefly, after exposure, cells were fixed in 4% paraformaldehyde and permeabilized with 1% Triton X-100 and 0.1% sodium citrate. Samples were then incubated for 60 min at 37 °C in the absence and presence of exogenous TdT and incubated with fluorescein-conjugated dUTP for repair of nicked 3′-hydroxyl DNA ends. Mean cell fluorescence of 10,000 cells and percentage of TUNEL-positive cells were assessed by flow cytometry for each condition.

### DNA fragmentation assay

MDA-MB-231 cells were treated with 7, 15 and 30 μg/ml of ZEO and positive control was treated with 5 μg/ml of etoposide. DNA extraction was performed by standard procedure as described before. After dissolving DNA in TE buffer, it was loaded on a 2% agarose gel and electrophoresis was performed for 2 hours and stained with ethidium bromide for DNA visualization.

### Reactive oxygen species (ROS) assay

ROS production was monitored by flow cytometry using 2′, 7′- dichlorodihydrofluorescin diacetate (DCFH_2_-DA) (Sigma-Aldrich). This dye is readily diffuses into cells and is hydrolyzed by intracellular esterase to yield 2′, 7′ dichlorodihydrofluorescin (DCFH_2_), which is trapped within the cells. Hydrogen peroxide or low molecular weight peroxides produced by the cells oxidizes DCFH_2_ to a highly fluorescent compound 2′, 7′-dichlorofluorescein (DCF). Thus, the fluorescence intensity was proportional to the amount of hydrogen peroxide produced by the cells. Briefly, MDA-MB-231 cells (1 × 10^5^ cells/well) were treated with IC50 concentration of ZEO for 12 h. Thirty minutes before the end of the experiment cells collected, centrifuged (1200 rpm, 5 min) and the pellet was washed with 1 mL of PBS, then the samples was treated with DCFH_2_-DA (20 μM) and kept in the dark. After 30 min the fluorescence was assessed by comparing two fluorescence excitation/emission 485–495 nm/525–530 nm using a flow-cytometer.

### Evaluation of mitochondrial membrane potential (ΔΨm)

To determine the changes in ΔΨm levels, the MDA-MB-231 cells were treated with 0–30 μg/ml concentrations of ZEO for 12 h in the same way as called above and incubated with 400 μl 50 μM Rhodamine 123, at 37 °C for 30 min. The cells were then collected, washed twice with PBS to remove extracellular Rhodamine 123. The fluorescent intensity of the Rhodamine 123 was measured by flow cytometry with excitation and emission at 488 and 525–530 nm, respectively. The mean fluorescence intensity represented the cellular levels of intracellular MMP.

### Caspase-3 activity assay

Caspase-3 activity was measured using colorimetric assay kit (BIOMOL International, USA), according to the manufacturer’s instructions. Briefly, MDA-MB-231 cells (1 × 10^6^) were treated with 0–30 μg/ml for 4 h and then each sample was harvested and washed with PBS. The cells were incubated with lysis buffer on ice and the protein concentration of each sample was determined using the Bradford method. Equivalent amounts of proteins for each sample were incubated with the appropriate caspase substrate and after 4 h, the absorbance at 405 nm was measured using a microplate reader.

### DNA oxidation analysis

Genomic DNA was extracted from MDA-MB-231 cells after 2 h treatment with 3, 7, 15, 30 µg/ml concentrations of ZEO along with untreated control according to standard phenol/chloroform extraction procedure. Same amounts of DNA (5 µg) was then digested with nuclease P1 (sigma N8630) and treated with alkaline phosphatase (NEB M0290S) and subjected to 8-oxo detection Elisa kit (Cayman chemical 589320) in total volume of 50 µl according to manufacturer’s protocol.

### Comet Assay

DNA strand breaks was measured by the alkaline comet assay, which is able to detect both single and double strand breaks in singled cells preparations^47^. Briefly, MDA-MB-231 cells (3.5 × 10^5^ cells/well) were treated with IC50 concentrations of ZEO for 2 h. Subsequently Cells collected, centrifuged (1200 rpm, 5 min) and the pellet was washed with PBS. Frosted microscope slides were dipped into hot 1% normal-melting-point agarose in PBS to the frosted area. The slides were air-dried and stored at room temperature until needed. A 200 μl of cell/agarose suspension (100 μl of cell suspension containing about 10^4^ cells was mixed with 100 μl of 1% low-melting-point agarose in PBS) was placed over the first agarose layer under a glass cover and incubated for 15 min in 4 °C to solidify. glass cover were removed, the slides were gently immersed in a cold lysis solution (2.5 M NaCl, 100 mM EDTA, 10 mM Tris-base, pH was adjusted to 10 with NaOH in 4 °C, 1% Triton X- 100 added just before use) for 1 h at 4 °C to lyse the cells. The microscope slides were then transferred to the horizontal gel electrophoresis unit filled with fresh, chilled electrophoresis buffer (denaturing buffer) (300 mM NaOH, 1 mM EDTA, pH 13) to a level of about 0.2 cm above the slides and left for 30 min in 4 °C to allow unwinding of DNA before electrophoresis. Electrophoresis was conducted for the next 30 min at 0.6 v/cm. The slides were then drained, placed on a tray and flooded slowly with neutralization buffer (0.4 M Tris, pH 7.5), for 5 min to remove alkali. Each slide were then stained with 100 μl 1X cyber green and kept in the dark for 30 min then covered with a coverglass and were imaged in fluorescent microscope (Axioscope 2 plus, Ziess, Germany). At least 100 comet images from each slide were examined. Images were evaluated by the open comet software and individual comet images analyzed for several features including tail length, percent of DNA in tail, tail moment and olive moment.

### DNA content and cell cycle phase distribution Analysis of MDA-MB-231 Cells Spheroids and Monolayer by Flow Cytometry

Monolayer (3.5 × 10^5^) MDA-MB-231 cells were treated with different concentration of ZEO (3, 7, 15, 30 μg/ml) for 4 and (3, 7, 15 μg/ml) for 24 h and MDA-MB-231 spheroids were treated with ZEO IC50 concentration (118 μ/ml) for 24 h. At the end of the treatment, cells were harvested, washed with 1 ml PBS, centrifuged at 1200 rpm for 5 min at 4 °C then fixed with ethanol 70% for 24 h in 4 °C. After incubation cells were centrifuged and ethanol was removed. The pellet was resuspended in PBS and incubated with 20 μg/ml RNAase (Sigma-Aldrich) and propidium iodide (20 μg/ml) (Sigma-Aldrich) in 37 °C in dark for 30 min. Finally, the distribution of different cell cycle phases was determined by flow cytometry and data analysis was carried out using the FlowJo software Version 7.6.1.

### UV-Visible spectroscopy

Rat hepatocyte double stranded DNA (dsDNA) was isolated by standard phenol-chloroform method. DNA stock solution was prepared in Na_2_HPO_4_-NaH_2_PO_4_ (0.1 M) buffer and stored at 20 °C. DNA concentration and quality was evaluated by NanoDrop (thermoscientific-USA) and UV-Visible spectrophotometer (Cary 100 Bio-model, Australia). UV-Visible spectra of ZEO were recorded using spectrophotometer in phosphate buffer (0.1 M with pH = 7.4) at room temperature over a wavelength range 190–600 nm. Initially the absorbance of ZEO concentrations ranging from 0 to 32 μg/ml in phosphate buffer at 298 K was measured, the solvent was taken as reference, and then similar procedure was carried out after addition of constant DNA concentration (50 μg/ml).

### Fluorescence experiments

Fluorescence experiments were carried out on a Carry eclipse (Australia) spectrophotometer. In the competitive binding studies, constant concentrations of DNA and Ethidium bromide (EB) (50 μg/ml and 2.6 μM, respectively) with increasing concentrations of ZEO (0–256 μg/ml) were used. ZEO emission spectra were recorded in the range of 530–700 nm upon excitation at 500 nm. The slit width was set as 10 nm/10 nm for ex/em, respectively. The spectroscopic experiments were performed at 310 K.

### CD spectral measurements

CD spectra of pure DNA and its complex with ZEO were recorded at room temperature with CD spectrophotometer (Circular Dichroism 215, Aviv, USA). Circular dichroism studies were carried out using ZEO concentration 16 μg/ml and final DNA concentration of 100 μg/ml. All spectra were recorded in far-UV range. Quartz cuvette with a path length of 1 cm was used for sampling. A spectrum of buffer solution was recorded and subtracted from the spectra of DNA and DNA-ZEO complex.

### Statistical Analysis

Experimental data processing was carried out using Microsoft Excel 2013 software and results were presented as mean ± standard deviation of three or more independent experiments. The significant differences between means were determined by t-test when statistical significance was P value ≤ 0.05.
